# Machine Learning Densities, Detonation Velocities, and Formation Enthalpies of Energetic Materials Using Quantum Chemistry Descriptors

**DOI:** 10.1021/acs.jctc.5c00865

**Published:** 2025-08-28

**Authors:** Patrick Kimber, James Mattock, Sophia Wheeler, John Mullaney, Alison Beardah, Justin Fellows, Kenny Jolley, Felix Plasser

**Affiliations:** † Department of Chemistry, 152008 Loughborough University, Loughborough LE11 3TU, U.K.; ‡ 502513 DSTL Porton Down, Salisbury, Wiltshire SP4 0JQ, U.K.

## Abstract

The prediction of detonation parameters is a challenging task requiring to bridge the gap between microscopic molecular features and macroscopic materials properties. Whereas traditional routes are concerned with empirical equations, we present a machine learning approach to this task here. Our approach capitalizes on molecular descriptors from high-level quantum chemistry as input to produce models fitted against experimental reference data to model three key quantities: the crystalline density, the detonation velocity, and the heat of formation. To determine the detonation products, we use a new nonempirical product optimization scheme, maximizing the heat release, which is extensible to any molecular composition. We find, for all three properties, that the machine-learned results significantly surpass standard rule-based schemes. Finally, we present an all *in silico* scheme for predicting detonation velocities, highlighting that this is almost as good as when experimental densities are used as input. In summary, we believe that this work is a major step toward the goal of accurately predicting detonation parameters by showing how to leverage the power of quantum chemistry for this task.

## Graphical abstract

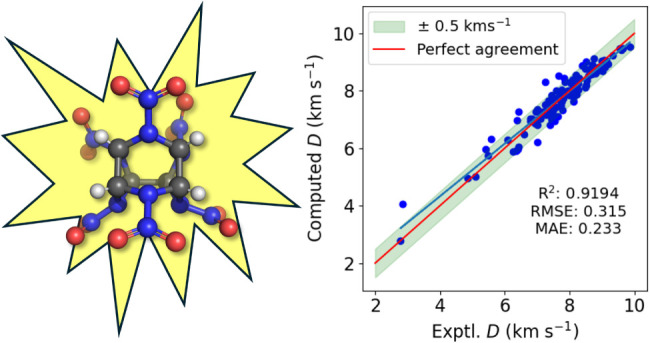

## 1 Introduction

Energetic materials, principally explosives, propellants, and pyrotechnics, have a wide range of uses, including both military and civilian applications.^1^ This has inevitably led to the design of such materials becoming a rapidly advancing field. Given the inherent complexities in the synthesis and analysis of energetic materials, the prediction of novel compounds is an ideal application for quantum mechanical methods.^2^ The varied applications of energetic materials have resulted in a significant body of work as different use-cases require radically different properties.^3,4^ Therefore, a method of accurately quantifying the properties of energetic materials is crucial. There currently exist a large array of methods and computational approaches, ranging from high-level single molecule approaches to machine learning.^5–8^

Computational approaches to modeling molecular structures and crystals have become an essential part of the materials discovery pipeline since they can be utilized as both a predictive and diagnostic tool. In particular, in the context of energetic materials, it is desirable to computationally screen new candidate molecules avoiding the need for specialized equipment and the risk that is invariably associated with experimental research on explosives. The detonation behavior of energetic materials is crucially influenced by their density and solid state enthalpy of formation, and these two properties have received specific emphasis in the literature.^9–13^ Well-established approaches, based around these two properties, for the prediction of detonation properties of energetics involve the use of thermochemical codes, the use of empirical models, and structure–activity relationships.^9,14,15^ Thermochemical codes generally require the density and enthalpy of formation as input parameters to determine detonation properties. For densities, empirical models have been developed starting from a single-molecule perspective^10^ and have been extended to include a consideration of the molecular environment.^11^ Empirical schemes for enthalpies of formation include a consideration of bond enthalpies or proceed via group additivity approaches.^12,13^ Finally, we note that a specific challenge in using empirical equations to compute detonation velocities is that these usually rely on an *a priori* determination of the detonation product distribution,^9,16,17^ a problem, which we will revisit below.

Identifying energetic molecules with high densities is often the main design strategy for energetic material discovery, since empirical schemes suggest that the density has the largest influence on detonation velocity and pressure.^9,18^ Densities can be trivially approximated by dividing the molecular mass by the molecular volume, however, such an approach neglects all influence of the crystalline environment. A partial improvement is given by group additivity approaches.^19^ However, these neglect information regarding molecular configuration and molecular connectivity meaning that not all isomeric structures would obtain distinct densities. Additionally, an explicit consideration of intermolecular forces is absent from group additivity approaches, and additivity schemes rely heavily on having a large amount of experimental reference data upon which to parametrize the atom and group volumes. As the availability and power of computational resources has increased, methods for simulating molecular crystals explicitly have become more readily accessible.^20–22^ These operate via the use of periodic density functional theory while cheaper methods based on force fields have also been developed.^23^ While these simulations can directly address the problems faced by additivity methods, they are not without their own challenges and even sophisticated crystal structure prediction routines can struggle to correctly identify the crystal structures of novel organic molecules.^24^ On the other hand, quantum chemistry computations on individual molecules are clearly cheaper; however in this case the challenge is to bridge between microscopic molecular properties and the macroscopic properties of interest.

In recent years, artificial intelligence (AI) and machine learning (ML) have emerged as attractive techniques to computational chemists, with applications in the development of new computational methods,^25–27^ molecular property prediction and discovery,^28,29^ and even the mapping of entire chemical landscapes for small organic molecules.^30^ More specifically, ML has been used for energetic materials properties. Densities have been modeled previously using features generated by cheminformatics toolkits such as RDkit.^31^ Going beyond scalar quantities (e.g., the oxygen balance), other featurization techniques have been developed to create input features by mapping structural information to vector quantities based on molecular connectivity or fingerprints.^32^ Other approaches for density ML models have considered molecular topology as a starting point for feature generation, using molecular graphs as inputs.^33^ The enthalpy of formation has been identified as an intuitive input parameter for detonation velocity models, however computing accurate values for this is an additional challenge. Recent efforts in this regard have utilized deep learning models for gas phase formation enthalpies,^34^ building upon existing ML methods for obtaining geometries, vibrational frequencies and energies from the generative GDB-11 database.^35–38^

A special challenge in the prediction of energetic materials properties is the scarcity of data, with detonation parameters available only for a few hundred molecules. This poses severe challenges in implementing a brute force big-data approach. Conversely, we opt here for a chemically informed procedure feeding in molecular properties, which are likely to be relevant to describe solid-state interactions. The numerical values for these properties, in turn, are determined by modern quantum chemistry methods. Our overall approach is outlined in Figure 1. We will focus on three materials properties, the density (ρ), the heat of formation (Δ*H*_f_), and the detonation velocity (*D*) and investigate how well they can be predicted based on quantum-chemistry data. First, we will investigate basic models for all three quantities and these will be described in Section 2 along with more general background information. These basic models all possess a fairly simple functional form based on only a small number of molecular features. To enable our ML approach, we will first augment the set of features by a variety of data points that can be obtained from quantum chemistry computations. These will then be fed into our ML models to obtain solid state corrections for our results and thus improved accuracy. The ML approach will be described in Section 3. Subsequently, a detailed discussion of the results obtained with various models will be presented in Section 4. During this work, we will also put specific emphasis on how to determine the products formed during explosion and what accuracy in terms of densities is required for a full *in silico* determination of the detonation velocity.

**Figure 1:**
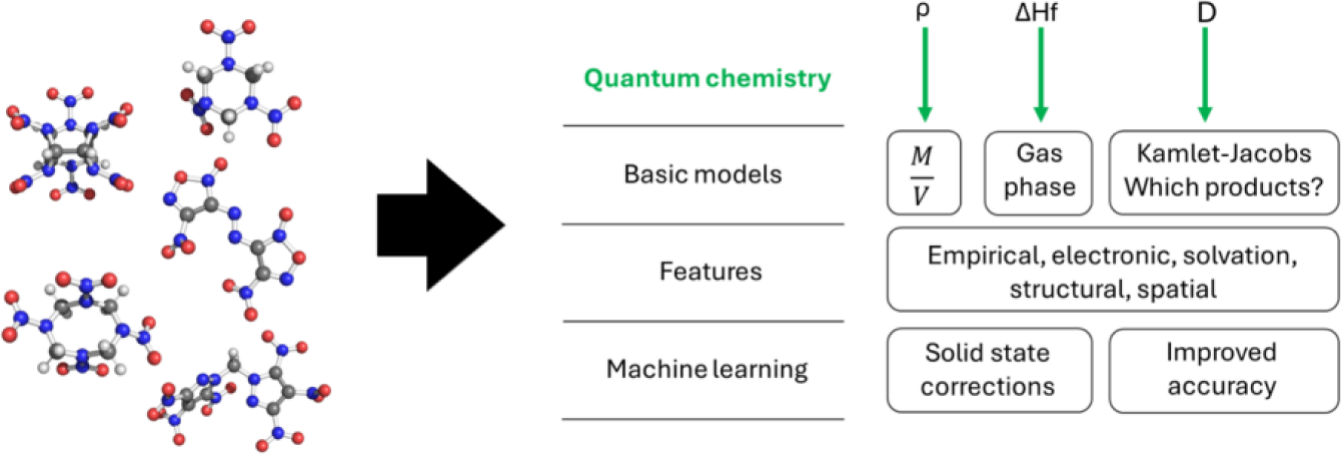
Flowchart of the procedure employed in this work to model the three parameters of interest: density (ρ), enthalpy of formation (Δ*H*_*f*_), and detonation velocity (*D*).

## 2 Background

### 2.1 Densities

Using isolated molecules as a starting point, the trivial approximation for the density relies on dividing the molecular mass (*M*) by the molecular volume (*V*).

1 $$\rho_{\mathrm{triv}}=\frac{M}{V}$$

This type of approach has been evaluated for a set of 180 energetic molecules by Rice et al., where the volume was computed at 0.001 electrons/bohr^3^ of the electron density.^10^ It was found that the root-mean-square prediction error was 3.7% with a maximum error of 0.178 g cm^–3^. Overall, this approach was found to perform poorly for molecules where extensive intermolecular forces, in particular hydrogen bonding, were expected in the crystalline phase.

If the crystal structure is known, then the true crystalline density can be obtained as

2 $$\rho =\frac{Z\times M}{V_{\mathrm{uc}}}$$

where *Z* is the number of molecules per unit cell. ρ differs from ρ_triv_ because the unit cell volume *V*_uc_ differs from the molecular volume *V*. *V*_uc_, in turn, is crucially affected by intermolecular interactions and potentially by voids in the crystal. In light of this, the single-molecule model was later extended by Politzer et al. to include corrections based on an analysis of the molecular electrostatic potential (ESP) as a way to estimate a molecule’s propensity to form intermolecular interactions in the solid state.^11^

### 2.2 Detonation Velocities

The detonation velocity, *D*, can be approximated via the Kamlet–Jacobs equation.^9^

3 $$D=A\phi^{1/2}\left(1+B\rho_{\exp}\right)$$

4 $$\phi =n_{\mathrm{gas}}\sqrt{{\mathrm{m̅}}_{\mathrm{gas}}q_{\mathrm{cal}}}$$

5 A=1.01,⁣B=1.30

where *A* and *B* are empirically derived coefficients, *n*_gas_ is the number of moles of gas produced per unit gram of explosive (g^–1^), *m̅*_gas_ is the average molecular mass of the gases produced (g mol^–1^), and *q*_cal_ is the heat of explosion (cal g^–1^). ρ_exp_ is the experimental loading density of the explosive (g cm^–3^); it enters eq 3 in highest order highlighting its importance in predicting detonation parameters. For maximal *D*, the loading density ρ_exp_ should ideally be equal to or very close to the material’s crystalline (theoretical maximum) density unless there are significant safety or stability issues with handling the material at this value.

While the density is a well-definited physical property of the material, the remaining terms in eq 3, from a theoretical standpoint, are traditionally determined according to rule-based schemes. The distribution of detonation products, which is a prerequisite for computing *n*_gas_, *m̅*_gas_, and *q*_cal_, is frequently determined for CHNO molecules based on the Kamlet–Jacobs (KJ) rules and proceeds as follows:

(1) Convert all H atoms to H_2_O­(g)
(2) Convert as many C atoms as possible to CO_2_(g)
(3) Convert remaining C atoms to C(s)
(4) Convert all N to N_2_(g)

The Kamlet–Jacobs rules are obviously limited to CHNO species. In addition, we highlight that these rules fail to account for some molecular compositions, for example, the absence of any oxygen atoms or an excess of oxygen remaining after step 2. Other rule based schemes have been conceptualized to account for more exotic molecular compositions. For example, the (modified) Kistiakowsky–Wilson (KW) rules can be employed depending on the oxygen balance of the structure; products for highly oxygen deficient structures are determined differently to those with an oxygen balance of more than −40%.^16,39^ An extension to the KW rules was later introduced by Springall and Roberts (SR) to redistribute the original products.^16,39^ More recently, Sivapirakasam et al. introduced a rule-based scheme to account for a wider range of molecular compositions including for compounds which contain metal atoms, are oxygen deficient, or contain halogens.^17^ Politzer and Murray evaluated the KJ, (modified) KW and SR rules for a set of 14 compounds with positive oxygen balances and found that the KJ rules provide product sets which yield the lowest errors in detonation velocity predictions using eq 3.^39^ More recently Muravyev et al. highlighted that the Kamlet–Jacobs rules for CHNO molecules generally result in product sets which optimize the enthalpy of explosion and, thus, the detonation velocity, obeying the so-called “maximal heat release principle”.^15^ In this work, we use the original Kamlet–Jacobs rules where possible but append two additional steps to account for the presence of excess oxygen, or halogen atoms:

(5) Convert remaining O to O_2_(g)
(6) Convert all X atoms to X_2_(g)

To summarize, a variety of schemes for determining detonation products have been developed. It is not always clear, which one to apply, especially if more exotic molecular compositions and elements other than CHNO are involved. We will revisit this problem below.

### 2.3 Enthalpy of Formation

In addition to the density, the solid state enthalpy of formation is sometimes used as an input parameter for modeling detonation properties of energetic materials, for example by thermochemical codes. A basic model for computing enthalpies of formation amounts to considering the general formation reaction

6 $$aC\left(s\right)+\frac{b}{2}H_{2}\left(g\right)+\frac{c}{2}N_{2}\left(g\right)+\frac{d}{2}O_{2}\left(g\right)+\frac{e}{2}X_{2}\left(g\right)\to C_{a}H_{b}N_{c}O_{d}X_{e}\left(g/s\right)$$

where, on the left side, all constituents except for solid carbon (graphite) are in the gas phase. The product on the right side can be chosen to be either gaseous or solid defining the gas phase [Δ*H*_*f*_(g)] and solid state [Δ*H*_*f*_(s)] enthalpies of formation. These are, in turn, related via the sublimation enthalpy:

7 $$\Delta H_{f}\left(s\right)=\Delta H_{f}\left(g\right)-\Delta H_{\mathrm{sub}}$$

In this work, we evaluate Δ*H*_*f*_(g) according to eq 6 by considering the total enthalpies obtained via DFT computations. To obtain Δ*H*_*f*_(s), we will later endeavor to include the sublimation enthalpy implicitly via our ML models.

Practically speaking all components of eq 6 except for solid carbon are easily amenable to quantum chemistry approaches. To obtain a reference value for carbon, we use 1/60 of the energy of C_60_ fullerene instead. This is certainly a crude approximation but we will show below that it is well-compensated via the ML schemes. Thus, in summary, Δ*H*_sub_ and the correction for C_60_ are both left to be corrected within the ML models.

## 3 Methods

Having discussed the overall background and state of the art, we now proceed to a description of our specific approach. We will first discuss the curation of our data set of experimental reference values before outlining specific details of the quantum chemistry computations performed to obtain our database of descriptors. Subsequently, we present our approach toward determining optimal product distributions. We will conclude with a discussion of the postprocessing steps and more technical details on machine learning and model evaluation.

### 3.1 Data Collection

Experimental reference data is somewhat scarce for energetic materials, particularly with regards to their detonation properties. In 2022, Muravyev et al. published an open data set of energetic materials containing detonation parameters for 260 CHNOFCl structures.^15^ The data set contains experimental (loading/charge) densities, theoretical maximum (crystalline) densities, solid state enthalpies of formation, detonation velocities and pressures, and heats of detonation. In this work, we consider only structures from the Muravyev data set if they are single molecule structures (no ionic structures or cocrystals) and solid under experimental conditions. Upon refining the Muravyev data set we retain 132 structures for which reference data is available. While reference values for the densities and detonation velocities are available for all of these structures, some values for the enthalpies of formation are absent. A selection of the studied molecules is shown in Figure 2. We found that some of the detonation velocity values from the Muravyev data set differ from other values published in the literature.^40–43^ These discrepancies and the final values used for our data set are discussed in more detail in Section S2.

**Figure 2:**
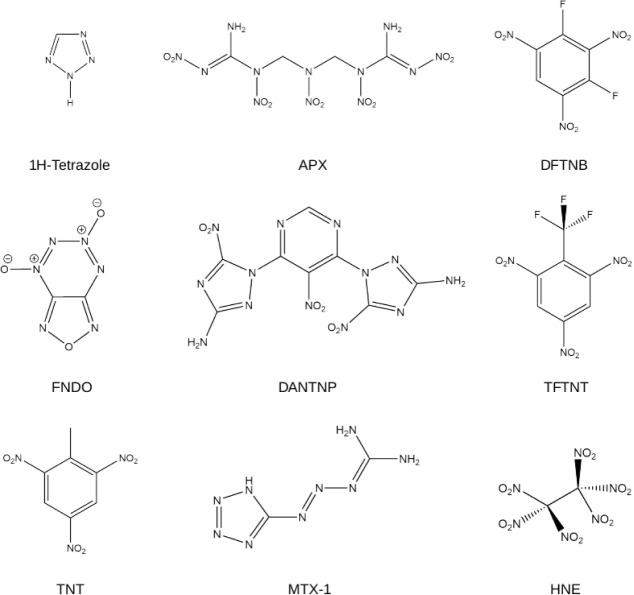
Selection of the 132 energetic molecules studied within this work.

Noting that the density is an elementary property of molecular solids, not specific to energetic molecules, we have constructed an additional data set using the Cambridge Structural Database (CSD).^44^ We have queried the CSD according to the following criteria in order to create a larger data set used for density modeling: First, the molecules must contain C, H, N, and O atoms, and may contain heavier atoms up to Cl but excluding Li, Be, Ne, Na, Mg and Al. Next, the molecule’s crystalline density was queried between 1.5 and 2.5 g cm^–3^, with a molecular mass no larger than 500 g mol^–1^. The entries must contain only single molecule structures; this excludes ions, cocrystals and solvates. We also exclude powder structures, structures with significant disorder, and structures which are labeled as having significant error. The R-factor for the entry must be less than 0.05. Finally, we only consider structures which have been added to the CSD since November 2020 in order to target newer structures or entries on the assumption these may have better accuracy than legacy entries. These criteria ensure that the millions of entries in the CSD are refined to a manageable number in terms of computational effort and data curation. After removing duplicate entries from the CSD along with all energetics from the first set, our query returned 801 unique molecules which will be used for our second density data set and these are described in the Supporting Information.

### 3.2 Quantum Chemistry Computations

In this section, we provide the details of the quantum chemistry calculations performed in order to obtain molecular descriptors. Geometry optimizations, vibrational analyses, and additional single-point computations were performed for all molecules in ORCA,^45^ using the r^2^SCAN-3c method.^46–49^ All structures were optimized and confirmed as being minimum energy structures via the vibrational analysis. Using the vibrational analyses, we compute the reactant total enthalpy (*H*) as well as the vibrational enthalpy (*H*_vib_) and entropy (*S*_vib_), as determined via the modified rigid-rotor harmonic oscillator (RRHO) model.^50^ Polarizabilities (α), dipole moments (μ) and quadrupole moments (*Q*) are computed for all molecules at their optimized geometries at the r^2^SCAN-3c level. Molecular volumes (*V*) and surface areas (SA) are determined by the GEPOL algorithm,^51^ where atoms are first represented with spherical surfaces corresponding to their van der Waals radii. The algorithm then uses a tessellation approach to select the parts of these spherical surfaces which form the molecular surface. In practice, these descriptors are obtained by running a single point conductor-like polarizable continuum model (CPCM) solvation computation^52^ and specifying dielectric constant = 1 and refractive index = 1. These descriptors are summarized in Table 1.

**Table 1:** Descriptors Used in This Work from ORCA DFT (r^2^SCAN-3c) Calculations^a^

Name	Descriptor
*H* (*H*_vol_)	Total enthalpy (per volume)
*E*_disp_	Dispersion energy
*H*_vib_	Vibrational enthalpy
*S*_vib_	Vibrational entropy
Δ*H*_*f*_(g)	Gas-phase enthalpy of formation, eq 6
*q*_cal_	Enthalpy of explosion (cal g^–1^)
SA (SA_vol_)	Molecular surface area (per volume)
*V*	Molecular volume
μ (μ_vol_)	Molecular dipole moment (per volume)
*Q* (*Q*_vol_)	Molecular quadrupole moment (per volume)
α (α_vol_)	Trace of molecular polarizability tensor (per volume)

a The subscript “vol”denotes that the volume normalised descriptor is also considered.

Aside from the above-presented descriptors, we aim to obtain a more detailed description of how the target molecule interacts with a condensed phase environment. For this purpose, we perform computations using the SMD solvation model.^53^ The motivation behind this approach is not to model a specific solvent but to learn how the molecule generally interacts with a condensed phase environment and to determine descriptors related to the individual interaction terms. Our assumption is if a molecule is strongly stabilized by a solvent due to electrostatic interactions or hydrogen bonds, then the same interactions may also operate in the solid state providing additional cohesive energy. SMD computations are performed in connection with the ωB97M-V/def2-TZVP level of theory^54,55^ as implemented in Q-Chem 6.0.^56^

The electrostatics modeled by the SMD solvation approach (see Table 2) are based on the electron density. The overall solvation energy can be partitioned into electrostatic (*E*_GENP_) and cavitation-dispersion terms (CDS). The GENP term depends on the dielectric constant ϵ of the solvent and we use a value of ϵ = 2 here to model a generic reaction field. In contrast, the CDS term is obtained as a response to other solvent properties. Of immediate interest in this work are the Abraham’s hydrogen bond acidity (SolA) and basicity (SolB) of the solvent. Within Q-Chem 6.0, the van der Waals radii of oxygen atoms are scaled according to SolA below a value of 0.5. In the interest of consistency, we perform a reference computation where SolA is set to 0.5, and the GENP term is taken from this computation. Then, SolA and SolB are independently increased to 0.93 and 0.5 respectively. The difference between the reference computation and the computations where SolA and SolB are “switched on” yields two cavitation-dispersion terms representing the response to hydrogen bond acidity (*E*_CDSA_) and hydrogen bond basicity (*E*_CDSB_).

**Table 2:** Descriptors Used in This Work from Q-Chem SMD (*ω*B97M-V/def2-TZVP) Calculations^a^

Name	Descriptor
*E*_CDSA_	Solvation—H-bond acidity term
*E*_CDSB_	Solvation—H-bond basicity term
*E*_GENP_	Solvation—electrostatic energy

a These descriptors are normalized by volume per default.

### 3.3 Optimal Product Distributions

Before we can proceed we need to address one crucial question. Some of the central parameters discussed above, such as *q*_cal_, *n*_gas_, and *m̅*_gas_ depend on the products formed. We discussed the standard rules in operation in Section 2.2. However, such empirical rule based schemes have two clear problems: (i) if competing schemes exist for a given molecular composition, then it is not clear which one to choose and (ii) such schemes are not available for all elements potentially of interest. To avoid this problem, here we introduce a nonempirical scheme for determining detonation products which is applicable to all molecular compositions without the need to adjust or modify steps. Our approach is to perform an optimization of products in order to maximize the enthalpy of explosion, thus obeying the “maximal heat release principle” per construction. We provide a mathematical formulation for this approach here.

The optimization approach begins by considering the general reaction equation for the detonation process

8 $$C_{b_{1}}H_{b_{2}}N_{b_{3}}O_{b_{4}}X_{b_{5}}...\to x_{1}H_{2}O+x_{2}{\mathrm{CO}}_{2}+x_{3}N_{2}+x_{4}X_{2}+...$$

where the left side represents the chemical structure of the target molecule and the right side represents all potential products formed. The composition of the reactant molecule can thus be summarized as a vector **b** and the product distribution as a vector **x**. Furthermore, we define a matrix **A** whose rows and columns represent the individual chemical elements and products, respectively. For example H_2_O is represented *via A*_21_ = 2/*A*_41_ = 1 and CO_2_ *via A*_12_ = 1/*A*_42_ = 2. The stoichiometry of eq 8 can now be represented via the matrix vector product

9 Ax=b

where any vector **x**, that is a solution to eq 9, will provide a properly balanced reaction when inserted into eq 8. In addition, we require that all elements of **x** are positive.

The enthalpy of explosion is given as

10 $$q_{\mathrm{cal}}=\frac{H_{\mathrm{prod.}}-H\left(C_{b_{1}}H_{b_{2}}N_{b_{3}}O_{b_{4}}X_{b_{5}}...\right)}{M}$$

11 $$H_{\mathrm{prod.}}=x_{1}H\left(H_{2}O\right)+x_{2}H\left({\mathrm{CO}}_{2}\right)+x_{3}H\left(N_{2}\right)+x_{4}H\left(X_{2}\right)+...$$

12 $$H_{\mathrm{prod.}}=c^{T}x$$

where, in the last step we have defined a vector **c** whose entries correspond to the enthalpies of the different possible products. These enthalpies derive from small-molecule gas-phase quantum chemistry computations. For carbon we again use C_60_ as a reference. While this is certainly an approximation, we also note that the possible alternative of using perfectly monocrystalline and defect-free graphite does not seem more justified in representing the products of a violent detonation process.

It can now be seen that minimizing *q*_cal_ is equivalent to minimizing **c** ^*T*^ **x**. We can now rephrase the optimization problem as the task of finding the vector **x** that minimizes **c** ^*T*^ **x** subject to the constraint that **Ax** = **b** and that all elements of **x** are non-negative, that is

$$\begin{array}{ll} c^{T}x\overset{x}{\to }\min\\ \mathrm{subject}\;\mathrm{to} & \{\begin{array}{l} \mathrm{Ax}=b\\ \forall i:x_{i}\geq 0 \end{array} \end{array}$$

This is a standard *linear programming* task for which a number of efficient implementations have been devised; here we use SciPy.^57^ We will evaluate the performance of this optimization scheme against the KJ rules in conjunction with eq 3, and subsequently feed the optimized product data into our ML models.

The described approach will work whenever eq 9 is well-defined and possesses a solution. In practice, this means that suitable potential detonation products for all relevant chemical elements have to be included in the procedure. Aside from this, the approach is completely general.

### 3.4 Postprocessing

To carry out the remaining steps we developed an in-house Python toolkit, EMProp, for data parsing, postprocessing, and machine learning. We explain in more detail the procedures used to extract and manipulate the data. The descriptors determined at this step are summarized in Table 3. The procedure begins by reading in the 3D geometry of the molecule using OpenBabel.^58^ The molecular mass (*M*) is extracted, alongside other descriptors which OpenBabel provides: the octanol–water partition coefficient (log *P*), the topological polar surface area (TPSA), the molar refractivity (*A*), and the number of rotors (*N*_rot_). The trivial density estimate (ρ_triv_) is obtained by dividing the molecular mass by the molecular volume (eq 1).

**Table 3:** Molecular Descriptors Generated in the Post-Processing Stage

Name	Descriptor
*N*_X_	No. of atoms of X (X = C, H, N, O, Cl, F, S)
*N*_NO_	Number of nitrogen + oxygen atoms
OB	Oxygen balance (eq 13)
*A*_HB_/*D*_HB_	Number of hydrogen bond acceptors/donors
*N*_rot_ (*N*_rot, vol_)	Number of rigid rotors (per volume)
*M*	Molecular mass
ρ_triv_	Trivial density (eq 1)
*A*	Molar refractivity
log *P*	Octanol–water partition coefficient
TPSA (TPSA_vol_)	Topological polar surface area (per volume)
*m̅*_gas_	Average *M* of gases produced by detonation
*n*_gas_	Moles of gas produced by detonation

Next, we extract the number of hydrogen bond acceptors (*A*_HB_) and donors (*D*_HB_) by querying the structure with SMARTS strings.^59^ We denote hydrogen bond acceptors as N or O containing functional groups (excluding the N atoms of pyrrole, nitro, or amide groups). We consider any nitrogen or oxygen atom with at least 1 H atom attached as a hydrogen bond donor. SMARTS matching is also used to obtain the number of C, H, N, O, F, Cl, and S atoms present in the structure (*N*_X_, where X = C, H, N, O, F, Cl, or S) We use the atom counts to compute the oxygen balance (OB):

13 $$OB=\frac{-1600}{M}\times \left(2N_{C}+\frac{N_{H}}{2}\right)-N_{O}$$

as well as the NO count (*N*_NO_), which is simply the sum of the number of N atoms and O atoms.

Next, the gas phase enthalpy of formation (Δ*H*_*f*_(*g*)) is determined according to eq 6, using the *ab initio* total enthalpies, *H*, from the previous section. Following this, the expected products of the detonation reaction are determined either through the rule-based KJ scheme or our optimization approach and from these we can deduce the number of gas molecules produced (*n*_gas_), the average molecular weight of these gases (*m̅*_gas_) and the total enthalpy of the products. The specific (weight-normalized) enthalpy of the reaction in calories per gram (*q*_cal_) is determined by subtracting the enthalpy of the reactant (*H*) from the enthalpy of the products and dividing by *M*, see eq 10.

In some cases, we normalize descriptors by *V* in order to transform extensive descriptors into intensive ones. For example dividing *M* by *V* (as in eq 1), which are both extensive descriptors, yields a new intensive descriptor in the form of a trivially computed density (ρ_triv_). We also create a descriptor based on the surface area to volume ratio (SA_vol_), as well as the topological polar surface area per unit volume (TPSA_vol_). The solvation descriptors, *E*_GENP_, *E*_CDSA_, and *E*_CDSB_ are normalized by *V* per default. Likewise, the *A*_HB_ and *D*_HB_ descriptors are also normalized since the effect of hydrogen bond accepting or donating groups would generally be expected to depend on system size. We also consider the volume-normalized total energy of the molecule, *H*_vol_, in addition to the raw value, since it is unclear how much an ML model can infer about system size from the total energy. In other cases, for example, electronic properties such as polarizabilities, dipole moments and quadrupole moments, the situation is less clear since vector quantities do not necessarily scale with system size. To this end, we consider both the raw and volume-normalized forms, adding α_vol_, μ_vol_ and *Q*_vol_ to our descriptor set. Finally we consider both the raw number of rotors in the molecule as well as the number of rotors per unit volume (*N*_rot, vol_).

### 3.5 Machine Learning

The *scikit-learn* package^60^ is used within EMProp to construct the machine learning models in this work. We reduce the dimensionality of our descriptor set by first eliminating highly correlated features according to Pearson correlation coefficients, and then perform principal component analysis—further details are provided in Section 4.2.1. We evaluate linear regression, ridge regression (RR), kernel ridge regression (KRR), and the multilayer perceptron regressor neural network (MLP-NN) models. For densities and detonation velocities, the MLP-NN model is selected, while for solid state enthalpies of formation, RR is used. Model parameters are selected via a grid search cross-validation procedure. For standard model evaluation, predictions are made using 5-fold cross-validation (using always an 80/20 split) with the parameters selected by the grid search procedure and we report averaged performance metrics for the training and test sets from each fold. Where the model is trained and tested on separate data sets, the performance metrics correspond to the values computed from the test data.

### 3.6 Model Evaluation

The metrics used to evaluate model performance in this work are summarized here. The simplest metric is the mean absolute error (MAE):

14 $$\mathrm{MAE}=\frac{1}{N}\overset{N}{\underset{i}{\sum }}\left|y_{i}^{true}-y_{i}^{pred}\right|$$

where the absolute error of the predicted $\left(y_{i}^{\mathrm{pred}}\right)$ from the true $\left(y_{i}^{\mathrm{true}}\right)$ values for a given quantity of interest is averaged across all N molecules. We categorize the prediction quality for densities and detonation velocities according to the individual absolute errors of the predictions. To categorize the quality of density predictions, we use ranges of absolute error following the work of Goh et al., who proposed that excellent, informative, and poor predictions are characterized by errors below 0.03 g cm^–3^, between 0.03 and 0.05 g cm^–3^, and above 0.05 g cm^–3^, respectively. For detonation velocity we consider an absolute error of 0.5 km s^–1^ to be acceptable.

In addition to the MAE, we compute the root-mean-squared error (RMSE):

15 $$\mathrm{RMSE}=\sqrt{\frac{1}{N}\overset{N}{\underset{i}{\sum }}{\left(y_{i}^{\mathrm{true}}-y_{i}^{\mathrm{pred}}\right)}^{2}}$$

which is the quadratic mean of the difference between the predicted and true values. The RMSE is more sensitive to outliers than the MAE. Finally, we compute the coefficient of determination, *R* ^2^, which represents the proportion of variation in the true values explained from the input features. *R* ^2^ is expressed through the residual sum of squares and the total sum of squares:

16 $$R^{2}=1-\frac{\sum {\left(y_{i}^{\mathrm{true}}-y_{i}^{\mathrm{pred}}\right)}^{2}}{\sum {\left(y_{i}^{\mathrm{true}}-{\mathrm{y̅}}^{\mathrm{true}}\right)}^{2}}$$

where *y̅*^true^ is the mean of the true values. The maximum value of *R* ^2^ is 1, implying that the RMSE vanishes and indicates that the input features are able to perfectly model the true values. As a technical note, it may be pointed out here that the Pearson correlation coefficient (*R*) would only be defined for a linear regression analysis whereas the coefficient of determination (*R* ^2^) can be generalized to general nonlinear regression via eq 16, which is why we use the latter here.

## 4 Results and Discussion

We first present results using the basic literature models described in Section 2 as a reference. Following this, we proceed to machine learning techniques discussing, first, feature selection and proceeding to a presentation of our best models. We conclude with an application demonstration studying a set of isomeric cyclobutane nitric esters.

### 4.1 Basic Models

Our starting point for the prediction of densities is to use eq 1 using the molecular volumes computed using the GEPOL algorithm as detailed in Section 3. The results are shown for the CCDC and Muravyev (energetic) data sets in Figure 3.

**Figure 3:**
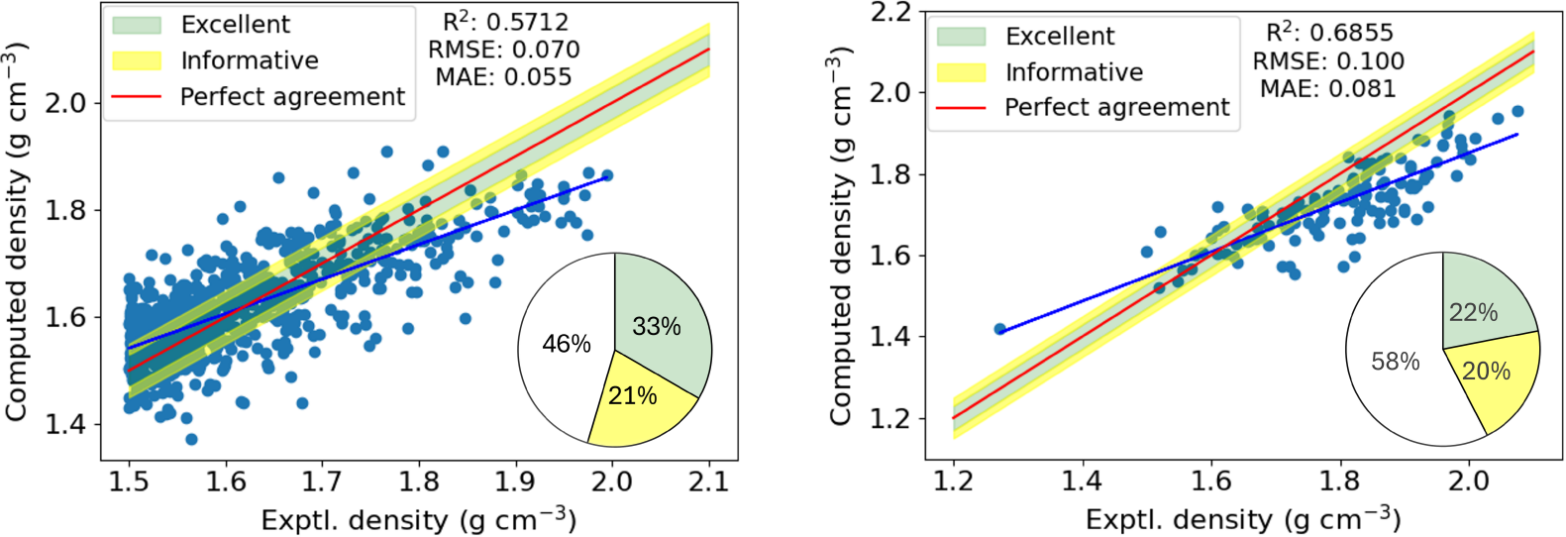
Trivial densities (eq 1) compared to the experimental densities for the 801 molecules extracted from the CCDC (left) and the 132 from the Muravyev data set (right). The red line indicates perfect agreement with the experiment, and the blue line is a regression line for our predicted values. The green and yellow shaded regions encompass absolute errors of ±0.03 g cm^–3^ and ±0.05 g cm^–3^ respectively, indicating excellent or informative predictions. The percentage of predictions within each shaded region are displayed in the pie charts.

For the CCDC data set, the coefficient of determination, R^2^ is 0.5725 and RMSE/MAE of 0.071/0.055 g cm^–3^ are obtained. We find that only 267 (33.3%) predictions are considered to be excellent, while 170 (21.2%) are considered informative. Nearly half of the predictions (364, 45.4%) are considered to be poor. Clearly this trivial model leaves plenty of room for improvement.

Moving from the general CCDC data set to the energetics, Figure 3 (right), further highlights the shortcomings of the trivial model. Over half of the predictions are found to be poor, while only 29 excellent (22.0%) and 27 (20.5%) informative predictions are made out of the 132 molecules in the data set. On inspection of Figure 3, we recognize that almost all of the poor predictions are underpredictions at higher densities, consistent with previous studies by Rice et al.^10^ The enhanced errors for the energetics data set are reflected in the increased error metrics (RMSE = 0.100 g cm^–3^, MAE = 0.081 g cm^–3^) compared to the CCDC data set.

Having evaluated a trivial approach to computing densities, we now move to assess the performance of eq 3 for computing detonation velocities. We use the experimental charge/loading densities of the energetic molecules at which the detonation velocity data is obtained. The remaining terms in eq 3 relate to the products of the detonation reaction. As a first option we determine these via the Kamlet-Jacobs rules as given in Section 2.2. The results are shown in the left-hand side of Figure 4. At a first glance, the empirical model seems to perform adequately for detonation velocity prediction, providing error metrics within the bounds of ±0.5 km s^–1^. However, on closer inspection one finds that detonation velocities above 5 km s^–1^ are systematically underpredicted. In total, there are 22 predictions with an absolute error of more than 0.5 km s^–1^, and of those, 20 are underpredictions.

**Figure 4:**
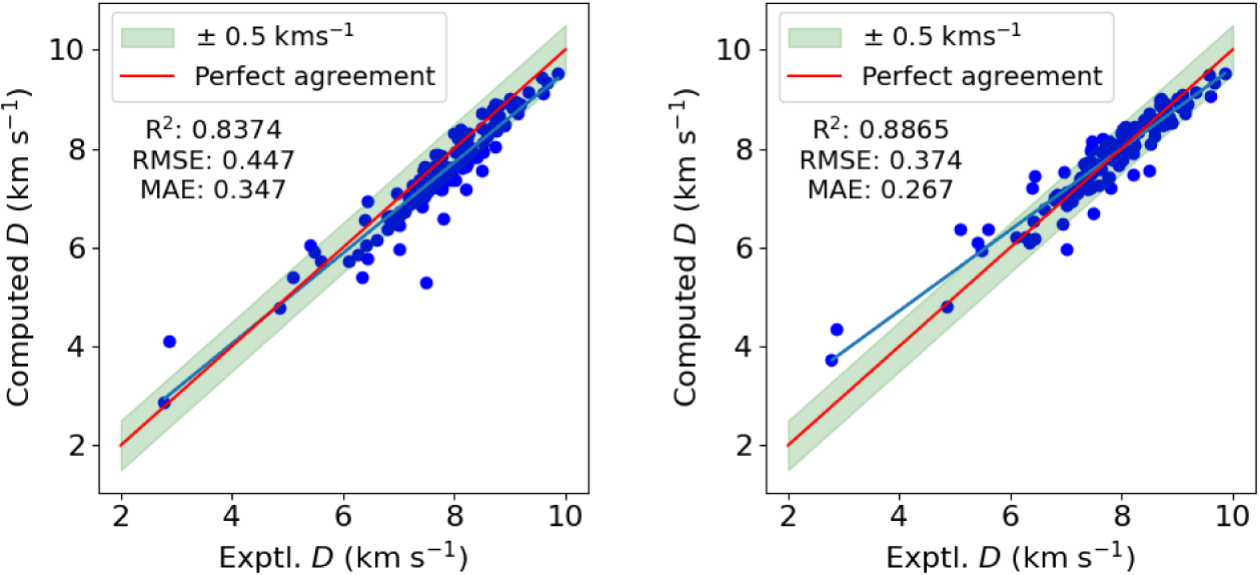
Computed detonation velocities (eq 3) compared to the experimental detonation velocities for the 132 molecules in the Muravyev data set, using the Kamlet–Jacobs rules (left) and our optimization scheme (right) for detonation products. The red line indicates perfect agreement with experiment and the blue line is a regression line for our predicted values. The green shaded region encompasses an absolute error of ±0.5 km s^–1^.

Considering the relatively poor performance of the results obtained in conjunction with the standard Kamlet–Jacobs rules, we have developed an alternative scheme with an intrinsically optimized product distribution, as explained in Section 3.3. The results, as shown on the right-hand side of Figure 4, are clearly improved in all relevant metrics. On inspection of the right-hand side of Figure 4, we recognize there are significantly fewer underpredictions in the detonation velocity above 6 km s^–1^ using the optimization scheme, in contrast to the rule-based scheme in the left-hand side of Figure 4. In total, there are 18 predictions with an absolute error of more than 0.5 km s^–1^. The improvement in the prediction of detonation velocities using eq 3 with the data from the product optimization scheme is reflected by the improved error metrics, with an *R* ^2^ of 0.8865, an RMSE of 0.374 km s^–1^, and an MAE of 0.267 km s^–1^. We can attribute this in part to the increased enthalpies of explosion computed with the product optimization scheme compared to the Kamlet–Jacobs’ rules, noting that changes to the distribution of products will have smaller effects on the remaining terms in eq 3 (moles of gaseous products and average gas weight). Furthermore, whereas previous work^15^ has denoted the Kamlet–Jacobs rules as attempting to obey the maximal heat release principle, we argue that our product optimization scheme does so per construction.

It is instructive to examine the largest underpredictions between the left and right-hand side of Figure 4 in order to discern the difference, in terms of product distribution and enthalpy of explosion, between the Kamlet–Jacobs rules and our product optimization scheme. The largest outlier using the Kamlet–Jacobs rules is TFTNT (trifluoro-trinitrotoluene, Figure 2) appearing at an experimental detonation velocity of 7.5 km s^–1^, which is 2.22 km s^–1^ higher than the predicted value. According to the Kamlet–Jacobs rules, TFTNT would decompose according to:

17 $$C_{7}H_{2}N_{3}O_{6}F_{3}\to 1H_{2}O+2.5{\mathrm{CO}}_{2}+4.5C\left(s\right)+1.5N_{2}+1.5F_{2}$$

and produces an enthalpy of explosion of 322.17 cal g^–1^. Conversely, our product optimization scheme finds that

18 $$C_{7}H_{2}N_{3}O_{6}F_{3}\to 3{\mathrm{CO}}_{2}+4C\left(s\right)+1.5N_{2}+0.5F_{2}+2\mathrm{HF}$$

resulting in a more than doubled enthalpy of explosion (754.14 cal g^–1^) and a vast improvement in the detonation velocity, underpredicted now by only 0.80 km s^–1^. The most notable difference in the product distributions is the absence of any water being formed but, instead, the hydrogen atoms are used to produce HF. Furthermore, not producing water produces an extra 0.5 mol of gaseous products (forms less C(s)). Therefore, in addition to the increased enthalpy of explosion, the product set from the optimization scheme also increases *n*_gas_/*m̅*_gas_.

The next largest underprediction (1.21 km s^–1^) is for DFTNB (difluoro-trinitrobenzene, see Figure 2) possessing an experimental detonation velocity of 7.8 km s^–1^. The Kamlet–Jacobs rules result in the detonation reaction:

19 $$C_{6}{\mathrm{HN}}_{3}O_{6}F_{2}\to 0.5H_{2}O+2.75{\mathrm{CO}}_{2}+3.25C\left(s\right)+1.5N_{2}+1F_{2}$$

which has an associated enthalpy of explosion of 689.00 cal g^–1^. Our product optimization scheme again suggests the formation of HF rather than water, resulting in an increased number of moles of gaseous products similar to the TFTNT case:

20 $$C_{6}{\mathrm{HN}}_{3}O_{6}F_{2}\to 3{\mathrm{CO}}_{2}+3C\left(s\right)+1.5N_{2}+0.5F_{2}+1\mathrm{HF}$$

Again, we find a strongly increased enthalpy of explosion (932.75 cal g^–1^). Using the data from the product optimization scheme, the error in the detonation velocity prediction is halved; underpredicted by only 0.60 km s^–1^.

As a final example, we compare the differences for DANTNP [bis­(amino-nitro-triazolyl)-nitropyrimidine, Figure 2], which in contrast to the previous molecules does not contain any halogen atoms. The experimental detonation velocity of DANTNP is 8.2 km s^–1^. Using the Kamlet–Jacobs rules, an enthalpy of explosion of 883.98 cal g^–1^ is obtained by the reaction:

21 $$C_{8}H_{5}N_{1}3O_{6}\to 2.5H_{2}O+1.75{\mathrm{CO}}_{2}+6.25C\left(s\right)+6.5N_{2}$$

and the detonation velocity is underpredicted by 1.03 km s^–1^ using this data. In contrast, our product optimization approach finds that

22 $$C_{8}H_{5}N_{1}3O_{6}\to 1.25{\mathrm{CH}}_{4}+3{\mathrm{CO}}_{2}+3.75C\left(s\right)+6.5N_{2}$$

produces a slightly larger enthalpy of explosion (951.50 cal g^–1^) which results in an improved detonation velocity prediction, underpredicted by only 0.72 km s^–1^. While the enthalpy of explosion is only around 70 cal g^–1^ larger using the optimization approach compared to the Kamlet–Jacobs rules, we identify that the production of CH_4_ rather than H_2_O is preferred, and allows the production of an increased amount of gaseous products and less C(s) overall. Therefore, the interplay of all the different terms which comprise ϕ in eq 3 can be identified to have a significant influence on detonation velocity predictions, even in spite of the density generally being considered the most important parameter.

To conclude our discussion of the basic models we show the results of computing gaseous enthalpies of formation against the reference solid state values in Figure 5. The presented results are DFT-computed values using a modern density functional along with thermostatistical corrections for vibrations and rotations but include no machine learning corrections or other parametrization. We generally find a reasonable correlation between theory and experiment. The mean absolute error between our gas-phase values and the experimental values is 143.9 kJ mol^–1^, while the RMSE is 173.5 kJ mol^–1^. The coefficient of determination is 0.8944. Surprisingly, the computed values are generally lower than the experimental solid state enthalpy of formation; according to eq 7, one might expect that our computed values would be greater than the solid state enthalpy of formation by the sublimation enthalpy. It is not immediately clear why this is the case, however we believe one contributing factor is that our “reference” value for the enthalpy of solid carbon is not that of graphite, but is instead obtained from a computation on gaseous C_60_, as discussed in Section 2.3. As a result, the associated total enthalpy of C(s) from eq 6 is larger than it would be for graphite, thus lowering our trivially computed gas phase enthalpy of formation. We will show below, that this contribution along with the sublimation enthalpy are readily accounted for within the ML models.

**Figure 5:**
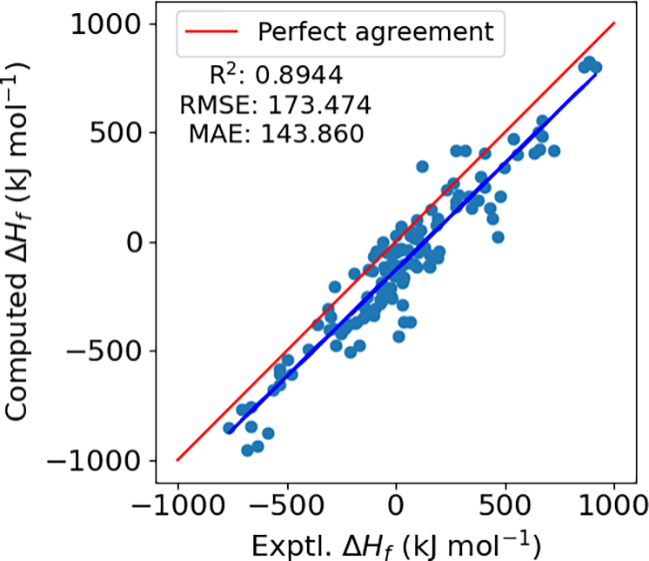
Gas-phase enthalpies of formation compared to the experimental solid state enthalpies of formation for the 124 molecules in the Muravyev data set. The red solid line indicates perfect agreement with experiment and the blue solid line is a regression line.

### 4.2 Machine Learning

Noting that there is clear room for improvement for all the above-presented literature approaches, we proceed to an evaluation of machine learning models. We first, briefly discuss our available descriptors and use a dimensionality reduction to obtain a set of linearly independent features. Subsequently we present cross-validated predictions on density, detonation velocity and enthalpy of formation. It will be of particular interest to evaluate whether all three can be modeled using all *in silico* approaches not requiring experimental inputs (other than the reference data).

#### 4.2.1 Feature Selection

In our attempt to bridge between properties of isolated molecules and macroscopic quantities it is first important to decide which features (i.e., molecular properties) are important. To do so, we compute the mutual Pearson correlation coefficients between all pairs of features as presented in Tables 1–3. We identify highly correlated features as those with a Pearson coefficient of more than 0.7, and where several features are highly correlated we keep only the one which is most correlated to the parameter of interest. For the detonation velocity and enthalpy of formation, all 41 descriptors from Tables 1–3 are considered. In addition, the experimental loading density is included for the former. For the density data set, we exclude *H*, *H*_*vol*_, *m̅*_gas_, *n*_gas_, *q*_cal_, Δ*H*_*f*_(*g*), as these are not expected to have any influence on the density, leading to 34 descriptors. Starting with these sets we are left with 15/22/20 descriptors for ρ/*D*/Δ*H*_*f*_(*s*) as summarized in Table 4.

**Table 4:** Descriptor Sets Used for Machine Learning the Three Quantities of Interest after Removing Highly Correlated Descriptors According to Their Pearson Coefficients

Target	Descriptor set
Density	*E*_CDSB_, *E*_GENP_, *H*_vib_, *S*_vib_, SA_vol_, *Q*_vol_, *N*_Cl_, *N*_F_, *N*_S_, *N*_NO_, OB, *D*_HB_, *N*_rot, vol_, ρ_triv_, log *P*
*D*	*E*_CDSA_, *E*_CDSB_, *E*_GENP_, *H*_vib_, Δ*H*_*f*_(g), μ, *Q*_vol_, α_vol_, *N*_C_, *N*_Cl_ *N*_F_, *N*_N_, *N*_NO_, OB, *A*_HB_, *D*_HB_, *N*_rot, vol_, *q*_cal_, TPSA_*vol*_, log *P*, *m̅*_gas_, ρ_exp_
Δ*H*_*f*_(s)	*E*_CDSA_, *E*_CDSB_, *E*_GENP_, *H*_vol_, Δ*H*_*f*_(g) μ, α_vol_, *N*_C_, *N*_Cl_, *N*_F_, *N*_H_, *N*_N_, *N*_O_, *N*_rot, vol_, *A*_HB_, *D*_HB_, *q*_cal_, TPSA_*vol*_, log *P*, *m̅*_gas_

Viewing Table 4, we note that, already at this stage, the density and Δ*H*_*f*_(s), are modeled with only *in-silico* descriptors. By contrast, we initially use one experimental quantity, the loading density (ρ_exp_), for modeling detonation velocities, as is common practice. However, we will later present a strategy of avoiding ρ_exp_ and, finally, will end up with pure *in-silico* strategies for all three quantities.

After excluding correlated variables via the correlation coefficients, we use principal component analysis (PCA) to perform further dimensionality reduction. We choose to select principal components which retain 99% of the explained variance. After scaling and performing principal component analysis, we obtain 13 principal components for densities, 17 for detonation velocities and 16 for enthalpies of formation. This will be used for the cross-validated predictions shown below.

#### 4.2.2 Cross-Validated Predictions

Within this work we evaluated the performance of four different regression models: Linear, Ridge, Kernel Ridge, and MLP-NN regression. After evaluating each model according to the metrics in Section 3.6, using the principal components from the previous section, the MLP-NN model was selected for densities and detonation velocities, while the ridge regression model was chosen for enthalpies of formation.

Having determined the optimal regression model for each parameter of interest, we now present the results of our predictions using the most successful machine learning model for each parameter of interest, using the principal components obtained from Section 4.2.1. For densities, we first consider the CCDC data set with cross-validated predictions made using the MLP regressor, shown in the left of Figure 6.

**Figure 6:**
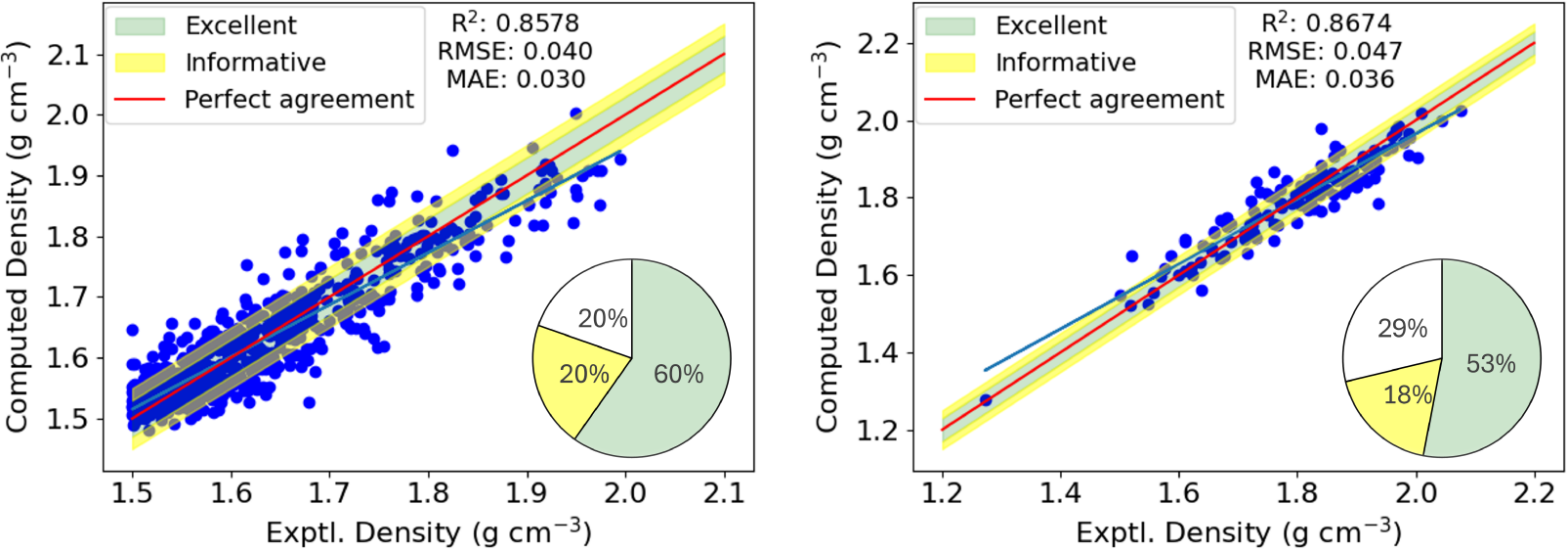
Cross-validated predicted densities using the MLP-NN model compared to the experimental densities for the two data sets. See caption of Figure 3 for details.

We find a dramatic improvement compared to Figure 3. In particular, there are significantly fewer overpredictions at low density, while in the high density regime the magnitude of the errors for underpredictions are much lower. The improved predictive power is also reflected by the improved performance metrics: the coefficient of determination, *R* ^2^ is increased from 0.5725 to 0.8577, the RMSE reduces from 0.070 to 0.040 g cm^–3^, and the MAE reduces from 0.055 to 0.030 g cm^–3^. Using the neural network approach, there are 479 (59.8%) excellent, 165 (20.6%) informative, and 157 (19.6%) poor predictions.

Next we show the results of the MLP-NN model for the energetics data set in the right-hand side of Figure 6. As with the CCDC data set, the results for the energetics data set are highly encouraging. The coefficient of determination is increased from 0.6855 for the trivial model to 0.8674 for our machine learning model. Moreover, the error metrics are reduced by more than half (RMSE = 0.047 g cm^–3^, MAE = 0.036 g cm^–3^) compared to Figure 3 (RMSE = 0.100 g cm^–3^, MAE = 0.081 g cm^–3^). For the energetics data set with the neural network model, there are 70 (53.0%) excellent, 24 (18.2%) informative, and 38 (28.8%) poor predictions. More generally speaking, the amount of underprediction in the higher density regime is significantly reduced when applying our neural network-based approach and making cross-validated predictions.

Finally, we train a model on the CCDC data set and use the Muravyev energetics data set as a test set on which predictions are made. The resulting correlation is shown in Figure 7. Overall, the agreement is quite good, with an *R* ^2^ much higher than our trivial model in Figure 3, and reduced error metrics. In comparison to the cross-validated predictions in Figure 6, the quality of the predictions in Figure 7 are slightly diminished, with an increase in the RMSE/MAE from 0.047/0.036 g cm^–3^ to 0.060/0.048 g cm^–3^. The coefficient of determination is also slightly reduced from 0.8674 to 0.7848. With regards to the prediction classifications introduced earlier, 44 predictions are excellent, 33 are informative and 55 are poor. We are pleased that our model trained on nonenergetic materials performs adequately for an energetic test set especially since previous approaches in this regard, found a significant drop off in the predictive ability of their models.^31^ But for practical application we note that, obviously, ML predictions on energetic materials will be improved if the training set does indeed contain energetics.

**Figure 7:**
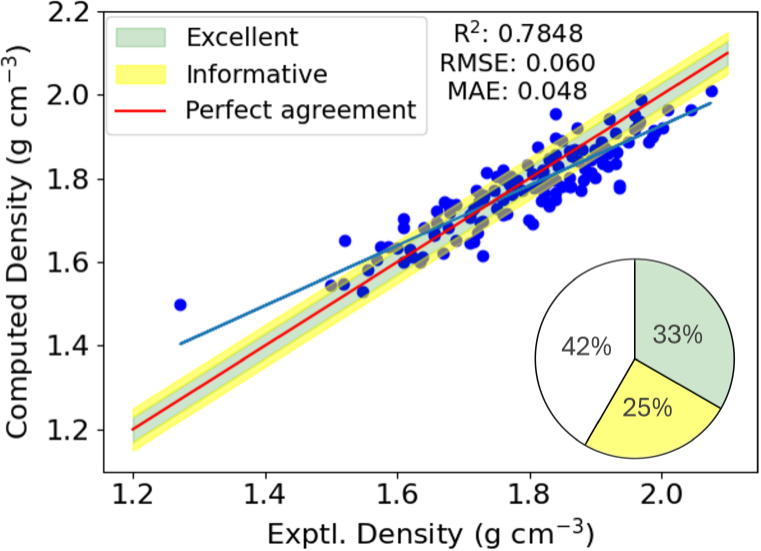
Predicted densities for the 132 molecules in the Muravyev data set using the MLP-NN regressor, trained on the 801 molecules in the CCDC data set (see caption of Figure 3 for details).

Next, we proceed to detonation velocities using, in the first instance the experimental loading density (ρ_exp_) as input. The cross-validated predictions of detonation velocities using the MLP-NN regressor are shown in Figure 8. At a first glance, our machine learning approach exceeds the performance of the empirical models afforded by the Kamlet–Jacobs equation (eq 3) shown in Figure 4. Using the already optimized version as a reference, the RMSE/MAE are further decreased from 0.374/0.286 to 0.285/0.197 km s^–1^. The value of *R* ^2^ is increased from 0.8865 to 0.9334. Only 9 predictions made with the neural network approach have an absolute error of more than 0.5 km s^–1^. Of these, the largest absolute error is 1.5 km s^–1^ for MTX-1 with an experimental detonation velocity of only 2.856 km s^–1^, shown on the very left in Figure 8. As discussed in Section 3.1, MTX-1 represents a somewhat pathological case with quite nonideal explosive behavior. Similar problems were discussed in ref 42 who report an average overestimation of *D* by 1.45 km s^–1^ when modeling MTX-1 for different densities and experimental setups. The absolute errors of the remaining 8 samples are all below 0.9 km s^–1^. Out of these, we find 7 overpredictions and, interestingly, only one underprediction.

**Figure 8:**
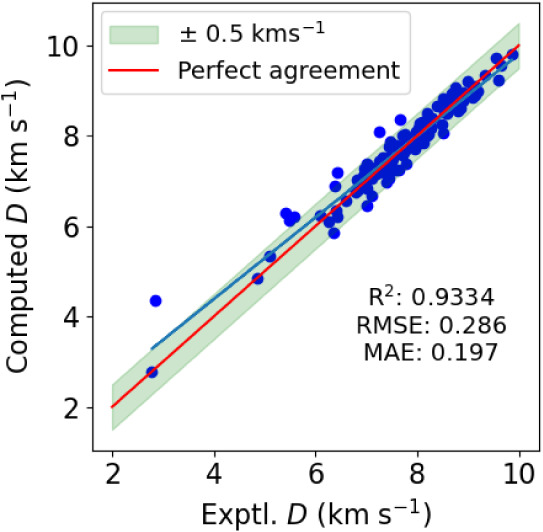
Cross-validated predictions of detonation velocities using the MLP-NN for the 132 molecules in the energetics data set using ρ_exp_ in the fit.The red solid line indicates perfect agreement with experiment and the blue solid line is a regression line.

The previous results used the experimental loading density (ρ_exp_) as input, which is common practice when fitting detonation velocities considering that it is also the one experimental quantity that goes into the Kamlet-Jacobs equation (eq 3). It is the purpose of this work to progress one step further and present an all *in silico* approach to the prediction of detonation velocities, not requiring any experimental input. This approach is, thus, applicable to completely unseen molecules. When developing such a model, we keep one stumbling block in mind. Detonation experiments are usually not performed at the theoretical maximum density (the crystalline density) but at a lower loading density. Which loading density to use is ultimately an experimental decision, that we cannot predict via our method. To represent this experimental decision, we use the fraction between the crystalline and loading density as an input parameter. When performing predictions on unseen molecules, this fraction also has to be specified. For example, we can specify to compute the detonation velocity for an unseen molecule at 95% of the theoretical maximum density.

Bearing this in mind, we can now move to a presentation of our all *in silico* results for the VOD, as shown in Figure 9. We find excellent agreement with almost no deterioration of the fit parameters compared to Figure 8, which used the experimental loading density in the fit. This highlights how our machine learning approach does indeed combine all the necessary information in this model. Compared to Figure 8, there is only a slight deterioration in *R* ^2^ (from 0.9334 to 0.9194) and also the error metrics are only slightly increased. There are now 13 predictions with absolute errors above 0.5 km s^–1^. Interestingly, MTX-1 is somewhat improved and overpredicted by only 1.2 km s^–1^. The second largest error is for BTATz [3,6-bis­(1*H*–1,2,3,4-tetrazol-5-ylimino)-1,2,4,5-tetrazine], overpredicted by 1.1 km s^–1^. All other errors lie within 0.9 km s^–1^ with, again, mostly overpredictions. Finally, reviewing Figure 4, we want to highlight that our all *in silico* model is still clearly better than using the Kamlet–Jacobs equation (eq 3), despite the latter using ρ_exp_ as experimental input.

**Figure 9:**
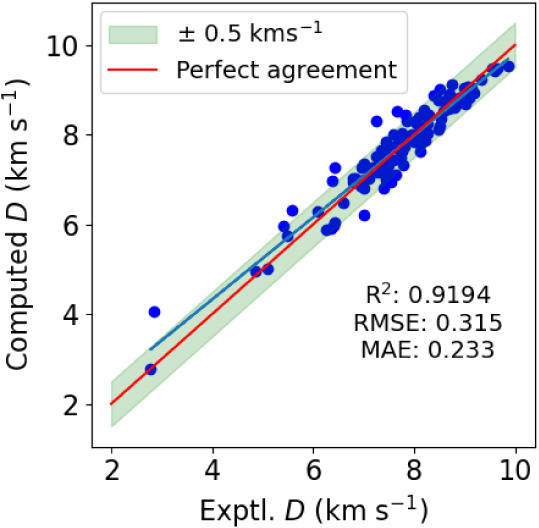
Cross-validated predictions of detonation velocities using the MLP-NN for the 132 molecules in the energetics data set using no experimental input data. The red solid line indicates perfect agreement with experiment and the blue solid line is a regression line.

Finally, we turn to our machine learning model for solid state enthalpies of formation, shown in Figure 10. In comparison to our trivial approach to modeling this property in Figure 5, we notice that there is significantly less underprediction for the solid state enthalpies of formation and overall a very good correlation. Earlier, we suggested that the error in the trivial model may have arisen from our choice to neglect the sublimation enthalpy from our model, and also as a result of our use of the gas-phase enthalpy of C_60_ as an approximation to obtain the value for solid carbon. Presently, it appears that we have been able to circumvent these limitations through the use of our machine learning model, as evidenced by the markedly improved predictions. The coefficient of determination, *R* ^2^ is 0.9457 while the RMSE and MAE are 79.0 and 52.1 kJ mol^–1^ respectively; a reduction of more than half compared to the trivial model.

**Figure 10:**
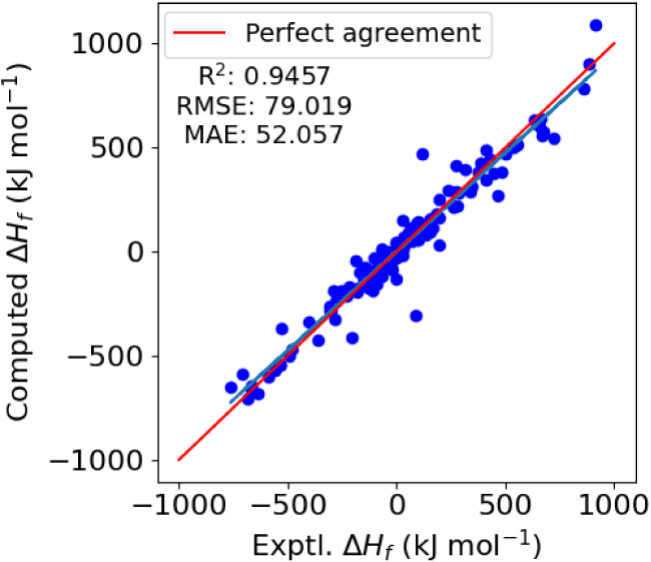
Cross-validated predictions of solid state enthalpies of formation using ridge regression for the 124 molecules in the energetics data set for which reference data is available. The red solid line indicates perfect agreement with experiment and the blue solid line is a regression line.

### 4.3 Application Demonstration: Cyclobutane Nitric Esters

In order to demonstrate the capabilities of our method, the machine learning model has been applied to a set of isomeric cyclobutane nitric ester derivatives as initially described by Barton et al.^61^ The molecular structures of the six molecules are presented in Figure 11. It was the goal of ref 61 to investigate how much these isomeric structures differ in their relevant properties. Before concluding, we want to briefly investigate our approach on this set of molecules. We do not use any experimental reference data on these molecules and will, now, test our purely *in silico* approach for predicting their properties.

**Figure 11:**
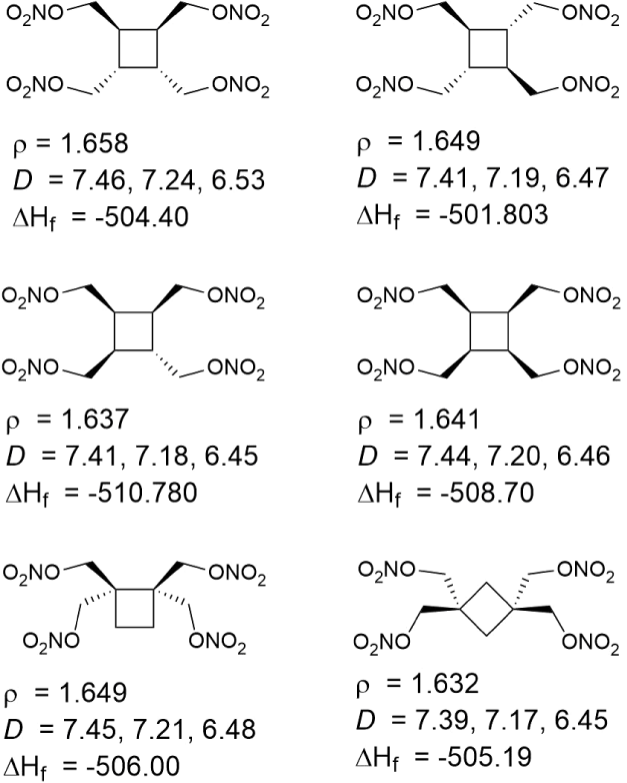
Depiction of cyclobutane nitric esters used to exemplify our *all in silico* prediction for densities (ρ, g/cm^3^), detonation velocities (*D*, km/s), and heats of formation (Δ*H*_*f*_ in kJ/mol). The three presented *D* values are given, in order, for a loading densities of 100%, 95%, and 80% of the theoretical maximum density.

Our model presents individual predictions on all six molecules. We find density predictions between 1.632 g/cm^3^ and 1.658 g/cm^3^. These are similar to the X-ray crystal densities reported in ref 61 only that our numbers show somewhat lower variability. Next, we present detonation velocities determined for 100%, 95%, and 80% of the theoretical maximum density. For results at 100%, we obtain values around 7.5 km/s, again very similar to ref 61. Using our model, we can also readily compute values at 95% and 80% of the maximum density, highlighting a signficant decrease in detonation velocity. Finally, the heats of formation are presented, all between 502 and 511 kJ/mol, yielding similar values to ref 61. In summary, we can conclude that our model readily produces very reasonable numbers for completely unseen molecules.

## 5 Conclusions and Outlook

We have highlighted modeling approaches for properties of energetic materials using extended quantum chemistry data as a starting point. The properties of interest were the crystalline density, the detonation velocity, and the solid state enthalpy of formation. We first evaluated trivial models before showing how one can construct machine learning models using descriptors from our quantum chemistry calculations as well as additional structural descriptors.

Starting with the density, the trivial mass per unit volume approach (eq 1) was generally found to provide only a very rough approximation, particularly for molecules with high densities (ρ > 1.7 g cm^–3^). This result is unsurprising given that the single-molecule picture neglects any information about the through-space interactions which would be present in the crystalline environment. Subsequent quantum chemistry computations were intended to produce descriptors reflecting the propensity of the molecule to interact with its environment. Indeed, using these feature sets combined with a multilayer perceptron neural network model, we found a significant improvement for the density data sets studied.

Before proceeding to detonation velocities, we have highlighted the drawbacks of rule-based schemes for determining detonation products; mainly that they are not a “one-size-fits-all” solution particularly where they are incompatible with particular molecular compositions. For example, an excess or lack of oxygen atoms in the case of the Kamlet–Jacobs rules, or molecules containing anything other than CHNO atoms. To overcome this problem, we have introduced a product optimization approach that maximizes the heat release during the explosion per construction. This scheme was shown to provide improved results in conjunction with the Kamlet–Jacobs equation while also providing a solid basis for our ML approach allowing to extend it to arbitrary molecular compositions.

Our predicted detonation velocities, using first the experimental loading densities as input, provided excellent correlation with an *R* ^2^ of 0.9334 and RMSE/MAE of 0.286/0.197 km s^–1^. We proceeded to an all *in silico* model not requiring any external input highlighting that it produced results of almost the same quality as when using experimental loading densities. Before concluding, we illustrated our method on a set of six isomeric cyclobutane nitric esters presenting all *in silico* predictions on these molecules.

Finally, we studied solid-state enthalpies of formation. The computed gas phase enthalpies already provided a reasonable correlation but, unsurprisingly, also showed a strong systematic shift. Proceeding to the ridge regression model, we found a vastly improved predictive model, with a 5% increase in *R* ^2^ to 0.9457, and around half the RMSE and nearly three times lower MAE.

In summary, we have highlighted the power of quantum chemistry in connection with ML models to predict energetic materials properties. We have shown a variety of quantum chemistry data that can be successfully used to augment the models to incorporate information about crystal packing and detonation dynamics. More specifically, we have highlighted the power of a new nonempirical product optimization scheme. We believe that these advancements presents an important step in the quest for improved materials predictions. In the future, it will be interesting to extend the presented approach to other detonation parameters as well as bulk materials properties, such as melting points.

## Supplementary Material





## Notes

Quantum chemistry and experimental data underlying the presented machine learning models (ZIP)
Further description of the data provided and notes on data curation (PDF)
