# Supplementary material for: Machine Learning Densities, Detonation Velocities, and Formation Enthalpies of Energetic Materials Using Quantum Chemistry Descriptors
Source: J Chem Theory Comput. 2025 Aug 28;21(17):8406–19. doi: 10.1021/acs.jctc.5c00865 (PMC12424169; doi:10.1021/acs.jctc.5c00865)
Supplement: Supplementary file 2 [file ct5c00865_si_002.pdf]

Supporting Information

for

Machine learning densities, detonation velocities, and formation enthalpies of energetic materials using quantum chemistry descriptors

Patrick Kimber,[1] James Mattock,[1] Sophia Wheeler,[2] John Mullaney,[2] Alison Beardah,[2] Justin Fellows,[2] Kenny Jolley,[1] Felix Plasser[1]

August 5, 2025

[1]Department of Chemistry, Loughborough University, Loughborough, LE11 3TU, U.K.
[2]DSTL Porton Down, Salisbury, Wiltshire, SP4 0JQ, U.K.

Contents

S1: Description of the developed dataset **2**

S2: Notes on data curation **3**

List of Tables

S1 Descriptors used in this work from ORCA DFT (r^2SCAN-3c) calculations. The subscript 'vol' denotes that the volume normalised descriptor is also considered. The right column indicates the variable name used inside the dataset. . . . 2

S2 Descriptors used in this work from Q-Chem SMD (ωB97M-V/def2-TZVP) calculations. These descriptors are normalised by volume per default. The right column indicates the variable name used inside the dataset. . . . 2

S3 Molecular descriptors generated in the post-processing stage. The right column indicates the variable name used inside the dataset. . . . 2

S4 Descriptors for experimental variables. The right column indicates the variable name used inside the dataset. . . . 3

S1: Description of the developed dataset

The below tables describe the dataset contained in the enclosed csv files with variable names as they are given in the csv files. Descriptors are described in more detail in the main text.

Table S1: Descriptors used in this work from ORCA DFT (r^2SCAN-3c) calculations. The subscript 'vol' denotes that the volume normalised descriptor is also considered. The right column indicates the variable name used inside the dataset.

Name	Descriptor	Variable
H (H_{vol})	Total enthalpy (per volume)	`E_tot` (`E_tot_vol`)
E_{disp}	Dispersion energy	`DispersionE`
H_{vib}	Vibrational enthalpy	`H_vib`
S_{vib}	Vibrational entropy	`S_vib`
$\Delta H_f(g)$	Gas-phase enthalpy of formation, Eq. (6)	`HOF`
q_{cal}	Enthalpy of explosion (cal g^{-1})	`Qcal_g`
SA ($\mathrm{SA}_{\mathrm{vol}}$)	Molecular surface area (per volume)	`SA` (`SA_vol`)
V	Molecular volume	`Volume`
μ (μ_{vol})	Molecular dipole moment (per volume)	`DipMom` (`DipMom_vol`)
Q (Q_{vol})	Molecular quadrupole moment (per volume)	`QuadMom` (`QuadMom_vol`)
α (α_{vol})	Trace of molecular pol. tensor (per volume)	`Polarizability` (`Polarizability_vol`)

Table S2: Descriptors used in this work from Q-Chem SMD (ωB97M-V/def2-TZVP) calculations. These descriptors are normalised by volume per default. The right column indicates the variable name used inside the dataset.

Name	Descriptor	
E_{CDSA}	Solvation - H-bond acidity term	`CDSA`
E_{CDSB}	Solvation - H-bond basicity term	`CDSB`
E_{GENP}	Solvation - electrostatic energy	`GENP`

Table S3: Molecular descriptors generated in the post-processing stage. The right column indicates the variable name used inside the dataset.

Name	Descriptor	Variable
N_{X}	No. of atoms of X (X=C, H, N, O, Cl, F, S)	`X_atoms`
N_{NO}	Number of nitrogen + oxygen atoms	`NO_count`
OB	Oxygen balance	`OB`
A_{HB} / D_{HB}	Number of hydrogen bond acceptors / donors	`HBA_vol` / `HBD_vol`
N_{rot} ($N_{\mathrm{rot,\ vol}}$)	Number of rigid rotors (per volume)	`rotors` (`rotors_vol`)
M	Molecular mass	`MW`
ρ_{triv}	Trivial density	`Dens_comp`
A	Molar refractivity	`MolRfrc`
$\log P$	Octanol-water partition coefficient	`logP`
TPSA ($\mathrm{TPSA}_{\mathrm{vol}}$)	Topological polar surface area (per volume)	`TPSA` (`TPSA_vol`)
$\overline{m}_{\mathrm{gas}}$	Average M of gases produced by detonation	`AvgGasWt`
n_{gas}	Moles of gas produced by detonation	`MolGasProd`

Table S4: Descriptors for experimental variables. The right column indicates the variable name used inside the dataset.

Name	Descriptor	Variable
ρ_{ch}	Loading/charge density	`ChDensity`
ρ_{exp}	Experimental crystalline density	`Dens_PK`
$\rho_{\mathrm{ch}}/\rho_{\mathrm{exp}}$	Ratio of loading vs experimental density	`Dmax`
D_{exp}	Experimental VOD	`VODexp`
$\Delta H_{f,\mathrm{exp}}(s)$	Experimental solid-state HOF	`HOF_exp`

S2: Notes on data curation

First, the detonation velocity for 1H-tetrazole (see Fig.2, main article) is given as 4.77 km s^{-1} at an experimental density of 1.51 g cm^{-3}. Other studies by Klapotke *et al.* and Jafari *et al.* have computed the detonation velocity of 1H-tetrazole with thermochemical codes, using the same density, and found a significantly larger value of over 7 km s^{-1}.[1, 2] Whilst computational codes may not produce perfect results, such a large discrepancy between these values along with a lack of experimental reports in the literature is unusual. To avoid such ambiguity, we have removed 1H-tetrazole from the dataset. Next, the detonation velocity for MTX-1 [2-(tetrazol-5-yl-diazenyl)guanidine], see Fig. 2) is given as 2.91 km s^{-1} at an experimental density of 0.90 g cm^{-3}. This is consistent with experimental work in the literature, however it has also been found that this molecule does not detonate at its crystalline density and that the detonation velocity is fairly insensitive to the density.[3] We opt to use the set of values (2.856 km s^{-1} at 0.64 g cm^{-3}) here since we can infer this represents a more efficient detonation process. Finally, the detonation velocity for [1,2,5]oxadiazolo[3,4-e][1,2,3,4]tetrazine-4,6-dioxide (FTDO) is given as 9.6 km s^{-1} at an experimental density of 1.55 g cm^{-3}. The primary source for this value is suspected to misreport the experimental density used and more recent studies have evaluated the detonation velocity of this molecule to only exceed 9 km s^{-1} at or near the theoretical maximum density of 1.85 g cm^{-3}.[4, 5] In light of this, we use the crystalline density value of 1.85 g cm^{-3} for FTDO and a detonation velocity of 9.25 km s^{-1}. A listing of the values we used is provided as Supporting Information.

References

[1] Thomas M. Klapötke, Marco Stein, and Jörg Stierstorfer. Salts of 1H-Tetrazole – Synthesis, Characterization and Properties. *Zeitschrift für Anorg. und Allg. Chemie*, 634(10):1711–1723, 2008.

[2] Mohammad Jafari, Kamal Ghani, Mohammad Hossein Keshavarz, and Fatemeh Derikvandy. Assessing the Detonation Performance of New Tetrazole Base High Energy Density materials. *Propellants, Explos. Pyrotech.*, 43(12):1236–1244, 2018.

[3] V. I. Kolesov, K. O. Kapranov, A. V. Tkacheva, and I. A. Kulagin. Explosive Characteristics of Tetrazene and MTX-1. *Combust. Explos. Shock Waves*, 57(3):350–355, 2021.

[4] Victor P. Zelenov, Nikita M. Baraboshkin, Dmitry V. Khakimov, Nikita V. Muravyev, Dmitry B. Meerov, Ivan A. Troyan, Tatyana S. Pivina, Alexandr V. Dzyabchenko, and Ivan V. Fedyanin. Time for quartet: The stable 3:1 cocrystal formulation of FTDO and BTF-a high-energy-density material. *CrystEngComm*, 22(29):4823–4832, 2020.

[5] Nikita V. Muravyev, Dominique R. Wozniak, and Davin G. Piercey. Progress and performance of energetic materials: open dataset, tool, and implications for synthesis. *J. Mater. Chem. A*, 10(20):11054–11073, 2022.
